# Epidemiology of Pediatric Functional Abdominal Pain Disorders: A Meta-Analysis

**DOI:** 10.1371/journal.pone.0126982

**Published:** 2015-05-20

**Authors:** Judith J. Korterink, Kay Diederen, Marc A. Benninga, Merit M. Tabbers

**Affiliations:** Department of Pediatric Gastroenterology and Nutrition, Emma’s Children’s Hospital Academic Medical Center, Amsterdam, The Netherlands; University Medical Center of Princeton/Rutgers Robert Wood Johnson Medical School, UNITED STATES

## Abstract

**Objective:**

We aimed to review the literature regarding epidemiology of functional abdominal pain disorders in children and to assess its geographic, gender and age distribution including associated risk factors of developing functional abdominal pain.

**Methods:**

The Cochrane Library, MEDLINE, EMBASE, CINAHL and PsychInfo databases were systematically searched up to February 2014. Study selection criteria included: (1) studies of birth cohort, school based or general population samples (2) containing data concerning epidemiology, prevalence or incidence (3) of children aged 4-18 years (4) suffering from functional abdominal pain. Quality of studies was rated by a self-made assessment tool. A random-effect meta-analysis model was used to estimate the prevalence of functional abdominal pain in childhood.

**Results:**

A total of 58 articles, including 196,472 children were included. Worldwide pooled prevalence for functional abdominal pain disorders was 13.5% (95% CI 11.8-15.3), of which irritable bowel syndrome was reported most frequently (8.8%, 95% CI 6.2-11.9). The prevalence across studies ranged widely from 1.6% to 41.2%. Higher pooled prevalence rates were reported in South America (16.8%) and Asia (16.5%) compared to Europe (10.5%). And a higher pooled prevalence was reported when using the Rome III criteria (16.4%, 95% CI 13.5-19.4). Functional abdominal pain disorders are shown to occur significantly more in girls (15.9% vs. 11.5%, pooled OR 1.5) and is associated with the presence of anxiety and depressive disorders, stress and traumatic life events.

**Conclusion:**

Functional abdominal pain disorders are a common problem worldwide with irritable bowel syndrome as most encountered abdominal pain-related functional gastrointestinal disorder. Female gender, psychological disorders, stress and traumatic life events affect prevalence.

## Introduction

Chronic abdominal pain is a common problem in childhood, with prevalence rates ranging from 0.3–19% in school-going children in the United States and Europe.[1] In almost 90% of these children, no explanatory organic cause can be identified.[2] Initially this condition was referred to as ‘recurrent abdominal pain’ RAP by Apley and Naish in 1957 and defined as “at least three episodes of abdominal pain, severe enough to affect their activities over a period longer than three months”.[3] In 1999 the pediatric Rome II criteria introduced the term abdominal pain related-functional gastrointestinal disorders (AP-FGIDs); which include functional dyspepsia (FD), irritable bowel syndrome (IBS), abdominal migraine (AM), functional abdominal pain (FAP) and functional abdominal pain syndrome (FAPS).[4] In order to meet these criteria symptoms had to occur weekly, persisting for over three months before diagnosis. With the introduction of the current Rome III in 2006 this criterion was redefined to persisting symptoms two months prior to diagnosis.[5]

Children with AP-FGIDs report significantly lower quality of life (QoL) scores compared to healthy peers and AP-FGIDs are ranked as second in causing school absence[6,7] In 29.1% of patients with chronic abdominal pain, pain persists even for more than 5 years, despite frequent medical attention.[8] Furthermore, functional abdominal pain disorders in childhood have a huge economic burden, as only the diagnostic workup is approximately 6000 dollar per child in the United States.[9]

The pathogenesis underlying AP-FGIDs remains unclear.[10] Altered gut motility, visceral hypersensitivity, abnormal brain-gut interaction, psychosocial disturbance and immune activation have been suggested as possible explanation for the symptoms.[11,12] Moreover, studies conducted in the United States and Europe reported that psychological symptoms, low socio-economic status, parental gastrointestinal complaints and single parent- and immigrant-households are associated with chronic abdominal pain in children.[1,13,14]

It has been commonly believed that functional abdominal pain disorders are a more evident problem in Western populations compared to developing countries. The purpose of the current study is to perform a systematic review and meta-analysis concerning the epidemiology of functional abdominal pain disorders in children worldwide in order to summarize the existing knowledge about its prevalence, geographic, gender and age distribution. In addition, we aim to review factors associated with functional abdominal pain disorders, such as psychosocial factors, quality of life, school absence, life events and socioeconomic factors.

## Methods

### Search strategy and study selection

The Cochrane Library, MEDLINE, EMBASE, CINAHL and PsychInfo databases were searched, up to February 2014. Studies on functional abdominal pain disorders were identified using the following terms: chronic or functional or recurrent abdominal pain, functional gastrointestinal disorder, stomach ache, abdominal migraine, irritable bowel syndrome or functional dyspepsia (both as medical subject heading (MeSH) and free text terms). These were combined, using the set operator AND, with epidemiology studies, identified with the terms ‘epidemiology, prevalence and incidence’ (MeSH and free text terms). A protocol of the current systematic review, including the full search strategy is provided in the S1 Protocol.

Abstracts were screened for eligibility. Potentially eligible studies were retrieved and read in full text to assess if they fulfilled all of the following inclusion criteria: (1) children aged 4–18 years; (2) with functional abdominal pain according to the ROME I, II, III criteria, Apley and Naish criteria or defined by the presence of nonorganic abdominal pain in children with at least three episodes of abdominal pain and/or weekly episodes of abdominal pain and/or a symptom duration of at least 3 months; (3) epidemiology studies of birth cohort, school based or general population samples and (4) results reported on epidemiology, prevalence or incidence. This screening was done by two reviewers (KD and JK) independently. Disagreement between the two reviewers was resolved by consensus when possible, or by consulting a third reviewer (MT) to made the final decision.

### Quality assessment

Because there is currently no gold-standard quality assessment tool for epidemiologic studies,[15] we composed a new assessment tool based on a scale for quantitative studies[16] and on a guideline for evaluating prevalence studies.[17] We screened for the following six criteria: (1) is method of subject selection described and appropriate? (2) Are subject characteristics sufficiently described, i.e. do they match the target population regarding to gender and age? (3) Is functional abdominal pain diagnosed appropriately? (4) Are the survey instruments reliable and valid? (5) Are the analytic methods described/justified and appropriate? And (6) were results reported in sufficient detail? Studies were scored to what extent they met each applicable criterion with: no, partial or yes.

### Data extraction

The following information related to data collection and results was extracted and entered into an Excel (Microsoft, Redmond, WA) spreadsheet: location, sampling strategy used to identify participants, sample size, age range, definition of functional abdominal pain disorders and the overall prevalence of functional abdominal pain disorders. If available, the gender, age and geographic distribution of the prevalence, socioeconomic factors, quality of life, psychosocial factors, school absence and life events were also reported.

### Statistical analyses

Meta-analysis methods were used to assess the prevalence of functional abdominal pain disorders. Either a fixed-effect model or a random-effect model was adopted to pool data according to heterogeneity. When the heterogeneity was significant, the random-effect model was applied, otherwise the fixed-effect model was used. Heterogeneity was calculated by a Cochrane *Q*-statistic, and the degree of heterogeneity was quantified by *I* ^*2*^ test.[18] *P*<0.10 in combination with *I* ^*2*^ >50% indicated significant heterogeneity.[19] Additionally, subgroup analyses were conducted to assess geographical, age and gender distribution of the prevalence. Sensitivity analyses were performed on validation of used criteria and questionnaire and on child or parental report of the functional abdominal pain disorder. Chi-square test was used to analyze age and gender associations, expressed as pooled odds ratio (OR) with 95% confidence intervals (CI). Level of significance was set at *p*<0.05. Publication bias was assessed by funnel plot and Egger's tests,[20] *p*<0.05 was considered to be statistically significant. All analyses were conducted using StatsDirect Medical Statistic Software (StatsDirect Ltd, Cheshire, England). Remaining results were reported in a descriptive way.

## Results

### Study selection and characteristics

**Fig 1:**
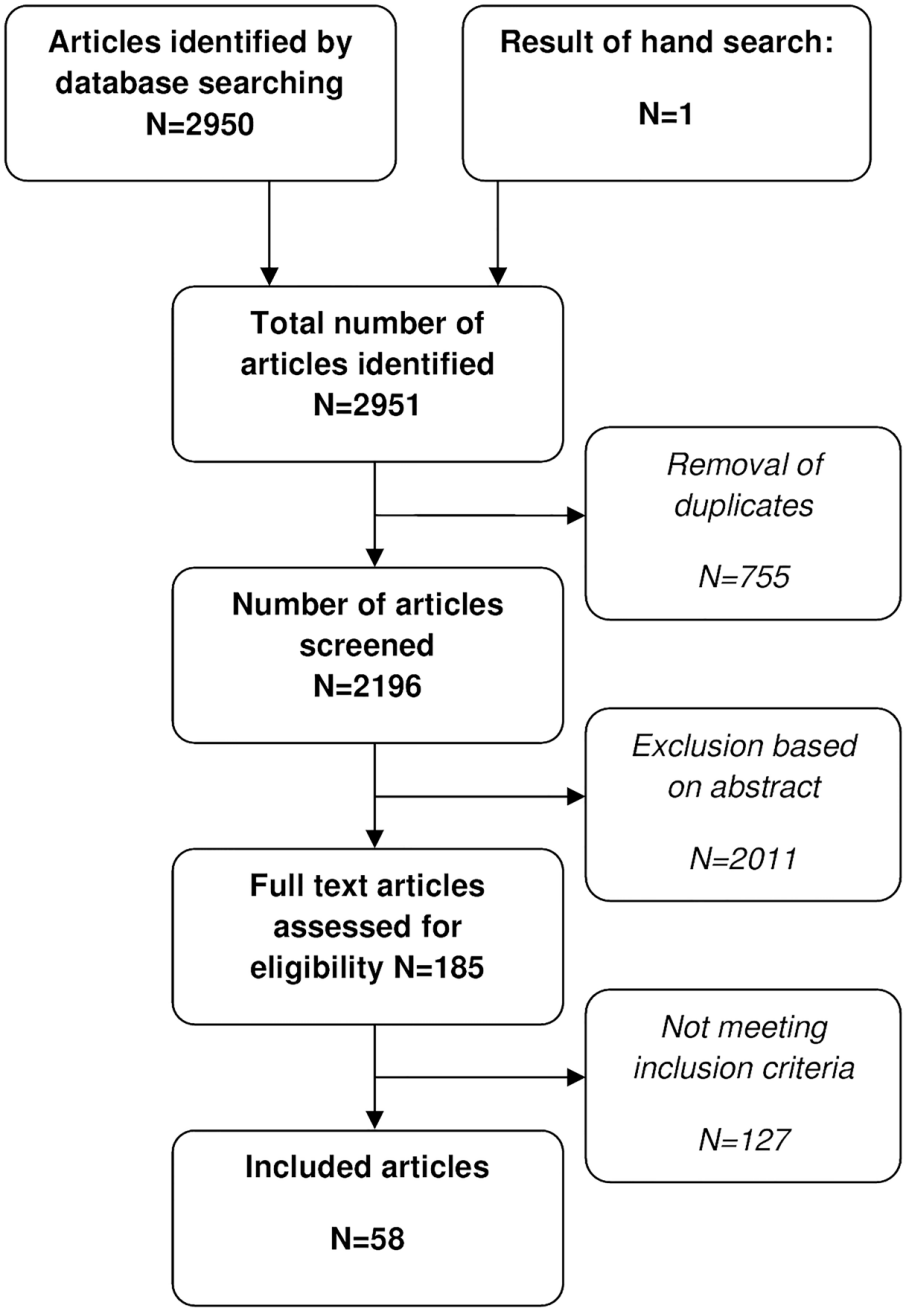
Flowchart showing results of literature search and study inclusion.

We found a total of 2196 titles and abstracts. After initial evaluation, 185 were judged potentially eligible. Finally, 127 articles did not meet our inclusion criteria. Reasons for exclusion were: adult study population (n = 42), not using appropriate definitions for functional abdominal pain (n = 26), irrelevant outcome measures/subject (n = 14), duplication of data (n = 18) and reviews, retrospective articles, abstracts or letter to the editors with insufficient information (n = 27). 58 articles remained,[3,6,14,21–75] including one systematic review (SR),[34] reviewing three articles[76–78] (Fig 1).

A total of 196,472 children with functional abdominal pain disorders were included with sample sizes ranging from 243[68] to more than 65,087.[34] Most children were recruited from school samples or birth cohorts.

Several different case definitions of functional abdominal pain disorders were used, including Apley and Naish (n = 18), ROME II (n = 11), ROME III criteria (n = 12) and self-reported functional abdominal pain disorders (n = 17). Different methodologies of data collection were used among studies. Questionnaires completed by parents and/or children were mostly used to asses functional abdominal pain data (n = 50). Other methods used were face-to-face interview (n = 6), clinical examination (n = 2) and a combination of questionnaire and interview (n = 4). In one single study and in the SR the method of data collection was not clear. Table 1 presents all study characteristics of the included studies.

**Table 1:** Characteristics of included studies.

*Study*, *year*	*Country*	*Population*	*Sample size(N)*	*Age range(years)*	*Method of data collection*	*Case definition*	*Prevalence(%)*
**Europe**							
Gulewitsch,[22]2013	Germany	School sample	1537	5–12y	Parental reports of QPGS-III	ROME II	7.7
Romero,[23] 2013	Spain	School sample	2575	8–16y	Child questionnaire	>3x AP in last 3 months	14.4
Luntamo,[25] 2012	Finland	School sample	2215	13–18y	Child questionnaire	Weekly AP in the last 6 months	5.6
Helgeland, [37]2010	Norway	Birth cohort	456	14y	Questionnaire	Apley and Naish	12.7
Rask,[40] 2009	Denmark	Birth cohort	1327	5–7y	Parental interview	Apley and Naish	7.6
Alfven,[42] 2008	Sweden	General cohort	2597	10–18y	Child and parental interview	AP weekly, for >6 months	19.8
Brun,[46] 2007	Sweden	School sample	1901	9–15y	Questionnaire^a^	last 3 months AP ≥ weekly	7.4
Ostberg,[47] 2006	Sweden	Welfare sample	5380	10–18y	Audio questionnaire	last 6 month AP ≥ 1 time a month	19.3
Bakoula,[74]2006	Greece	Birth cohort	7925	7y	Parental questionnaire	AP ≥ weekly	4.1
Dalh,[73] 2005	Denmark	School sample	849	9–13y	Parental questionnaire	ROME II	12.2
Tindberg,[49]2005	Sweden	School sample	695	9–13y	Child/parent questionnaire	Apley and Naish	12.7
Kokkonen,[69]2004	Finland	School sample	404	10–11y	Parental questionnaire and clinical examination	Apley and Naish	15.8
Groholt,[75] 2003	ScandinaviaIceland	General cohort	6040	7–17y	Child/parent questionnaire	AP weekly or every 2 weeks	8.3
Petersen,[53]2003	Sweden	School sample	1121	6–13y	Child/parent questionnaire	AP weekly ≥ 6 months	19.1
Bode,[51] 2003	Germany	School sample	1143	5–8y	Parental questionnaire^a^	Apley and Naish	2.5
De Giacomo,[55] 2002	Italy	School sample	808	6–12y	Child/parent questionnaire	ROME II	8.8
Harma,[54] 2002	Finland	School sample	15965	mean 15y	Questionnaire by students	AP weekly,≥ 6 months	10.0
Perquin,[59] 2000	Netherlands	School sample	4459	4–18y	Child/parent questionnaire	> 3 months AP	2.6
O'Donohoe,[61] 1996	UK	School sample	640	4–13y	Parental questionnaire	Apley and Naish	14.2
Abu-Arafeh,[62] 1995	Scotland	School sample	1754	5–15y	Questionnaire and interview	Symon and Russell	3.3
Mortimer,[70] 1993	UK	General cohort	1083	3–11y	Structure interview	Apley and Naish	8.4
Lundby,[72] 1990	Denmark	School sample	648	9–12y	Questionnaire	Apley and Naish	15.4
Faull,[71] 1986	UK	School sample	439	6y	Parental questionnaire/interview	Apley and Naish	25.1
Christensen,[63] 1984	Denmark	School sample	2530	5–16y	Questionnaire	Apley and Naish	11.4
Apley,[3] 1958	UK	School sample	1000	3–15y	Mother/child interview	Apley and Naish	10.8
**North America**							
Stanford,[45] 2008	Canada	General cohort	2271	12–13y	Child/parent questionnaire	AP weekly in the last 6 months	19.8
Youssef,[6] 2008	USA	Longitudinal Study in Adolesc. Health	20735	13–18y	In-home interview children	2–3 episodes/week the last 12 months	14.0
Malaty,[67] 2007	USA	School sample	925	4–15y	Questionnaire	AP >3 months continuous, interfere daily life	24.0
Uc,[68] 2006	USA	Annual school physicals	243	4–17y	QPGS-RII^a^ + clinical evaluation	ROME II	1.6
Hyams,[14] 1996	USA	School sample	507	12–16y	Bowel disease questionnaire^a^	Weekly AP in the last year	15.0
Sharrer,[64] 1991	USA	School sample	250	8–12y	Questionnaire parents	Apley and Naish	10.0
**South America**							
Saps,[21] 2014	Colombia	School sample	373	8–14y	QPGS-RIII^a^	ROME III	12.1
Silva,[35] 2011	Brazil	Birth cohort	1462	7–11y	Questionnaire	RAP for > 3 months, interfering daily life	21.6
**Asia**							
Sagawa,[24] 2013	Japan	School sample	3976	10–17y	QPGS-RIII^a^	ROME III	12.8
Phavichitr,[26]2012	Thailand	School sample	1181	12–19y	QPGS-RIII^a^	ROME III	24.0
Song,[27] 2012	Korea	School sample (girls)	820	12–17y	Child/parent questionnaire	ROME II	12.8
Zheng,[28] 2012	China	School sample	668	mean 14,8y	IBS Inventory^a^	ROME III	4.6
Zhou,[29] 2012	China	School sample	1362	12–18y	Questionnaire	ROME III	14.8
Devanarayana,[30] 2012	Sri Lanka	School sample	1365	13–18y	QPGS-RIII^a^	ROME III	17.8
Park,[79] 2011	Korea	School sample	1877	15–18y	IBS Module^a^	ROME III	19.0
Liu,[34] 2011	China	SR	65087			ROME II	4.6–23.4
Zhou,[36] 2011	China	School sample	3671	12–18y	Questionnaire	ROME III	20.0
Devanarayana,[31] 2011	Sri Lanka	School sample	2163	10–16y	QPGS-RIII^a^	ROME III	12.4
Endo,[33] 2011	Japan	School sample	2312	14–15y	ROME II modulaire questionnaire, self-reporting IBS questionnaire^a^	ROME II	15.4
Devanarayana,[32] 2011	Sri Lanka	School sample	428	12–16y	QPGS-RIII^a^	ROME III	13.7
Zhou,[38] 2010	China	School sample	2013	10–18y	Questionnaire	ROME III	20.7
Devanarayana,[43] 2008	Sri Lanka	School sample	734	5–15y	Parental questionnaire	Apley and Naish	10.5
Son,[44] 2008	Korea	School sample, (girls)	405	15–18y	unclear	ROME II	25.7
Dong,[48] 2005	China	School sample	5043	6–18y	Questionnaire	ROME II	14.2
Oh,[50] 2004	Singapore	School sample	3590	6–17y	Questionnaire	Apley and Naish	23.4
Boey,[52] 2003	Malaysia	School sample	1971	12y	Questionnaire and interview by pediatrician	Apley and Naish	23.1
Boey,[56] 2001	Malaysia	School sample	1462	9–15y	Interview by pediatrician	Apley and Naish	11.0
Boey,[57] 2001	Malaysia	School sample	1488	5–15y	Questionnaire and interview by pediatrician	Apley and Naish	9.6
Reshetnikov,[58] 2001	Siberia	School sample	449	14–17y	Bowel disease questionnaire^a^	ROME II	20.0
Boey,[60] 1999	Malaysia	School sample	148	11–12y	Parental questionnaire	≥ 3 episodes of AP for ≥ 3 months least	41.2
**the Middle East**							
Demirceken,[39] 2010	Turkey	Cohort general practitioner	250	5–18y	Questionnaire by child, parent and physician	ROME III	31.2
Sohrabi,[66] 2010	Iran	School sample	1436	14–19y	Questionnaire	ROME II	4.1
Telmesani,[41] 2009	Saudi Arabia	School sample (boys)	316	12–18y	Questionnaire	Apley and Naish	17.4

QPGS-RII/III; Questionnaire on pediatric gastrointestinal symptoms based on Rome II/III,

^a^Validated questionnaire

Due to significant heterogeneity a random effect-model was applied for all meta-analyses.

### Methodological quality assessment

We assessed the selection of study subjects. In 20 out of 58 studies they were not randomly selected out of a population sample. In 16 studies subjects did not match the target population appropriately, because for example only girls[27] or boys[41] were included, or because age range was limited.[33,37,45,52,69,71,74] In the majority of trials validated instruments were not used (n = 41). A detailed overview of the quality scores of all individual studies is listed in S1 Appendix.

### Prevalence

In general, pooled prevalence for functional abdominal pain disorders was 13.5% (95% CI 11.8–15.3). The reported prevalence ranged widely, from 1.6% to 41.2%. The forest plot of these data is shown in S2 Appendix. The funnel plot was symmetric and Egger’s linear regression test was not significant, which gives no indication for publication bias. Nineteen out of 58 studies reported prevalences of subtypes within AP-FGIDs (Table 2). Indication for publication bias was shown for meta-analyses of FD (Egger’s test *p* = 0.02).

**Table 2:** Pooled prevalence of functional abdominal pain disorders according to criteria used to define its presence, validation status of questionnaire, child/parental report, subtypes of AP-FDIG and geographical location.

	Number of studies	Number of subjects	Pooled prevalence (%)	95% CI	Heterogeneity	
					*I* ^*2*^	P value for *I* ^*2*^
***All studies***	*58*	*196*,*472*	*13*.*5*	*11*.*8–15*.*3*	*98*.*6*	*<0*.*001*
***Criteria used to define abdominal pain***						
** Self reported criteria**	17	77,980	13.2	10.2–16.6	99.4	<0.001
** Apley and Naish**	18	20,176	12.9	9.9–16.2	97.7	<0.001
** Rome II**	11	78,989	12.2	9.3–15.5	99.0	<0.001
** Rome III**	12	19,327	16.4	13.5–19.4	96.6	<0.001
***Validation status of questionnaire***						
** Validated**	17	21,809	11.9	9.0–15.2	98.0	<0.001
** Not validated**	41	174,663	14.1	12.1–16.3	99.3	<0.001
***Report***						
** Parental report**	12	15,639	11.0	7.4–15.2	97.9	<0.001
** Child report**	25	77,929	13.7	11.8–15.7	98.2	<0.001
***AP-FGID subtypes***						
** IBS**	16	28,399	8.8	6.2–11.9	98.6	<0.001
** FD**	9	11,516	4.5	1.2–9.9	99.2	<0.001
** FAP**	7	10,085	3.5	1.8–5.6	95.8	<0.001
** AM**	9	12,922	1.5	1.0–2.1	83.8	<0.001
** FAPS**	4	7,322	0.9	0.5–1.5	76.8	0.005
***Geographical location***						
** South America**	2	1,835	16.8	8.6–27.0	N/A	N/A
** Asia**	22	102,213	16.5	14.6–18.5	98.1	<0.001
** The Middle-East**	3	2,002	15.8	2.8–36.4	98.7	<0.001
** North America**	6	24,931	13.4	9.4–17.9	97.1	<0.001
** Europe**	25	65,491	10.5	8.3–12.8	98.8	<0.001

N/A; not applicable, too few studies to assess heterogeneity

Pooled prevalence numbers according to the different criteria used to define its presence, validation status of the questionnaire and the differences between child and parental report are shown in Table 2. Highest prevalence rates were found when using the ROME III criteria. The pooled prevalence of functional abdominal pain disorder was almost identical between studies that used a validated, compared to a non-validated questionnaire, or when functional abdominal pain was reported by children compared to parents (Table 2). The sensitivity analyses for Rome II, self-reported criteria and validated questionnaires were subject to publication bias calculated by Egger’s test, *p* = 0.04, *p* = 0.04 and *p* = 0.02 respectively.

#### Geographic distribution

**Fig 2:**
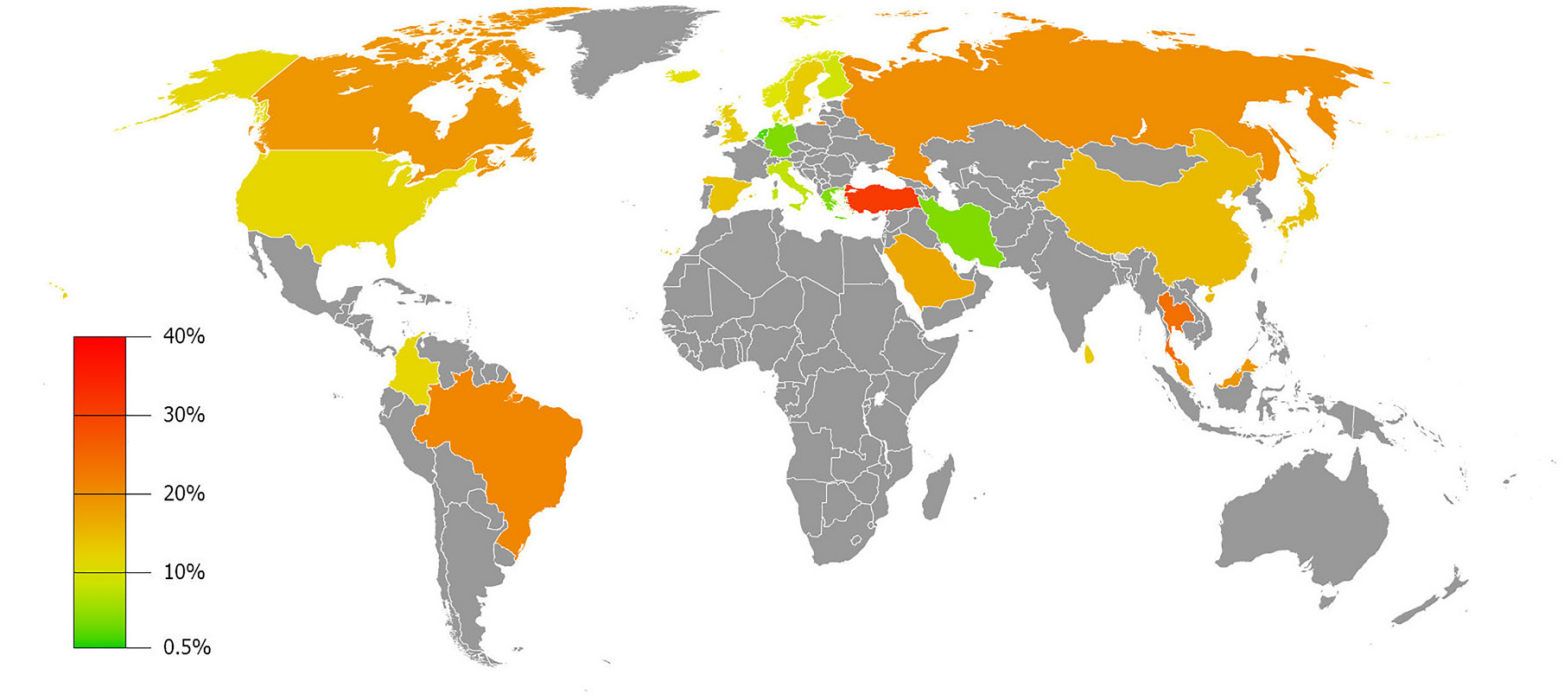
Geographic distribution of functional abdominal pain in children, presented in pooled-prevalence rates.

The majority of studies were conducted in Europe and Asia. A few studies were performed in the Middle East, North- and South America and prevalence data for Africa and Australia are currently lacking. The pooled prevalence of functional abdominal pain disorders subdivided for each continent is provided in Table 2. The prevalence rates did not differ extremely, with lowest prevalence occurring in Europe (10.5%) and the highest in South America (16.8%). Publication bias was only shown for meta-analysis of Europe (Egger’s test *p*<0.01). The prevalence per individual country studied is shown in Fig 2.

#### Gender prevalence

Gender prevalence was reported in 24 studies. All, but two studies,[51,70] reported a female predominance. After pooling data, a significant higher proportion of functional abdominal pain disorders among girls compared to boys was seen (15.9% vs. 11.5%, pooled OR 1.5, 95% CI 1.3–1.7, *p*<0.01). There was no evidence for publication bias by Egger’s test.

#### Age distribution

Relationship between age and functional abdominal pain prevalence has been evaluated in 36 studies. Because different age groups were used, we were unable to pool data for all single ages separately. Therefore, data were pooled for children <12 years and ≥12 years. No significant difference was found for the prevalence of functional abdominal pain disorders in children younger than 12 years as compared to children ≥12 years old (12.4% vs. 13.8%, pooled OR 0.9, 95% CI 0.5–1.4, *p* = 0.62). There was no evidence for publication bias (*p =* 0.26).

### Associated factors

#### Psychological disorders and Quality of life

Several studies reported an association between psychological factors with functional abdominal pain disorders.[22,25,28,36–38,44–46,48,54] Anxiety and depression were reported significantly more frequent among children with functional abdominal pain disorders compared to healthy children.[33,44,48,65] Furthermore one study showed that abdominal pain was a predictor of depression in 14–16 years old adolescents (girls: OR 2.4, 95% CI 2.0–2.9; boys: OR 2.3, 95% CI 1.7–3.1),[54] and vice versa, one study reported that depressive symptoms predict functional abdominal pain (OR 2.4; 95% CI 1.1–5.1).[37]

Two studies used the Strengths and Difficulties Questionnaire to screen for psychological problems, which is a 25 item-questionnaire, divided into five scales: hyperactivity—inattention, emotional symptoms, conduct problem, peer problem, and prosocial behavior scales.[22,25] Compared to children without abdominal pain, AP-FGIDs were associated with conduct problems (OR 4.1, 95% CI 2.8–5.9),[25] which especially concerned IBS patients (*p*<0.05).[22]

Eighty point five per cent of children with RAP reported school absence, at least one day during the third term of the year, compared to 44.6% of the healthy control group (*p*<0.01).[43] Furthermore, compared to controls, IBS patients showed significantly lower quality of life (QoL).[33,65] Park et al. investigated QoL with the World Health Organization QOL Scale, a 26-item questionnaire, divided into 5 subscales.[65] On each subscale (ranging between 1–5) children with IBS scored significant lower compared to non-IBS children (*p*<0.01): physical health (3.12 vs.3.42), mental health (2.94 vs. 3.11), social relationships (3.08 vs. 3.19), environment (3.07 vs. 3.18) and overall aspects (3.06 vs. 3.37).

#### Stress and Negative life events

Numerous studies showed an increase in prevalence of abdominal pain in children with high stress levels.[26,27,33,44,60,64,65] Measured on a 5-point scale (0 = never, 5 = always), 6.3% girls with mild stress (≤1.7 points) reported IBS, which significantly increased to 20.3% in girls with severe stress (>2.1 points).[27] In addition, mean total stress scores were significantly higher in the IBS group (119.7/200, SD 31.4) compared to healthy controls (95.9/200, SD 34.9, *p* = 0.03), measured on a 40 items Feel Bad Scale.[60] Similarly, patients with functional abdominal pain disorders reported significantly more traumatic- or negative life events.[30,31,50,80]

Twelve point two per cent of children with AP-FGIDs experienced the death of a close family member compared to 7.7% in the control group (*p<*0.02).[31] Also children with AP-FGIDs reported more frequent punishment by parents (6.7% vs. 3.8%, *p =* 0.04), frequent domestic violence (5.6% vs. 2.9%, *p =* 0.03), parental job loss (5.2% vs. 2.4%, *p* = 0.01) and hospitalization for another illness (16.3% vs. 9.5%, *p*<0.01).[31] Furthermore, any form of abuse was associated with an increase in the prevalence of functional abdominal pain. AP-FGIDs were significantly higher in those children exposed to sexual abuse (35.3% vs. 17.3%, *p* = 0.01), physical abuse (19.7% vs. 12.6%, *p<*0.01), and emotional abuse (27.4% vs. 16.9%, *p*<0.01).[30]

#### Socioeconomic status

Although a lower family income and low-educated families appeared to result in a higher percentage of children experiencing functional abdominal pain disorders, in most studies this trend was not statistically significant.[22,26,27,31,49,60,67,75] Malaty et al. reported the prevalence of RAP in different socioeconomic geographical areas, based on percentage of children receiving free or reduced-price school lunches. Low-income areas did not show a higher prevalence of RAP compared to high-income areas (23% vs. 27%, *p* = 0.38). On the other hand, a contrary finding was described by Groholt et al., who measured family income as the family's monthly disposable income and divided this into quartiles. RAP was reported in 6.6% in the highest quartile compared to 12.1% in the lowest quartile (*p*<0.01).[75] In the same study parental education was assessed. The prevalence of RAP was 7.8% among children living in low educated families (< 9 years education) compared to 9.5% in high educated families (> 12 years education), which was not significant.[75]

## Discussion

This is the first systematic review focusing on the prevalence of functional abdominal pain disorders in Western populations and developing countries. Our systematic analysis of available studies shows a worldwide prevalence of pediatric functional abdominal pain disorders of 13.5%, with approximately comparable rates across the continents. Irritable bowel syndrome (IBS) was the most often reported subtype of the abdominal pain related functional gastrointestinal disorders (AP-FGID). Higher prevalence rates were seen using the ROME III criteria and associations were shown with female gender, anxiety and depressive disorders, stress and traumatic life events.

Our findings are in line with a previous systematic review of Chitkara et al., which reported a high prevalence of childhood recurrent abdominal pain in Western countries.[1] We found a large variation in prevalence across studies, ranging from 1.6% to even 41.2%. This might be due to the variable age groups studied, the different definitions used to classify functional abdominal pain and different type of questionnaires used. However, sensitivity analyses did not reveal large differences in prevalence numbers (Table 2). The occurrence of abdominal pain reported by children was slightly higher compared to the occurrence reported by their parents. However, this concordance was expected to be less, since agreement between child and parents reporting pain and somatic symptoms is moderate and parents are likely to underestimate their child's pain.[81,82] Furthermore, since the publication of the pediatric Rome criteria for AP-FGIDs in 1999, higher pooled prevalence rates were found regarding studies using these strict AP-FGID criteria, up to a prevalence of 16.4%. A Sri Lankan population study even showed that the pediatric Rome III criteria were able to diagnose FGIDs more comprehensively than Rome II.[32]

The lowest prevalence of 1.6% was reported by Uc et al., though only African American children were included.[68] Other studies conducted in the USA showed a higher prevalence, ranging from 10–24%. The highest prevalence (41.2%) was reported in a small Malaysian study, including 148 children from a rural area. The authors suggested that this was due to the high prevalence of intestinal parasites in rural Malay school children.[83] In developing countries the prevalence of parasitic infections might be higher owing to potentially limited access to clean water, however, a Sri Lankan study identified parasitic infections as organic cause for RAP in only 7.7%.[80] Indeed literature shows that an association between AP-FGIDs and amebiasis is questionable.[84,85]

Prevalence rates range widely between countries. In addition to methodological differences, this may arise from factors such as diverse cultural, dietary, genetic, environmental conditions and different health care systems. Turkey showed the highest prevalence of functional abdominal pain disorders which was based on a small sample of 250 children. A prevalence of FD of 31% was reported, which is considerably higher than the total pooled prevalence of FD (4.5%, Table 2).[39] An explanation for this high prevalence could be that children were not screened for Helicobacter pylori which might result in an overestimation. In 65% of Turkish children presenting with recurrent abdominal pain and dyspepsia an infection with H.pylori can be found. [39]

According to different continents, the pooled prevalence was more stable, though was slightly lower in European studies and generally higher in studies from South-America and Asia. This finding is in line with the observation that the Rome III criteria were able to diagnose FGIDs more comprehensively than Rome II, since most Asian studies were only recently conducted and as a result used these criteria.[32] Moreover South-America and Asia are upcoming economies, with a change in (fast)food habits, a higher expectation from children, particularly towards their school achievements,[44] and consequently higher levels of stress.

In accordance with earlier data a predominance of functional abdominal pain disorders was found in girls.[1] This dominance in girls was reported in all different continents across the world. It has been suggested that levels of sex hormones might play a role, which is supported by observations that premenopausal patients present with exacerbation of their abdominal pain symptoms at time of menses.[86] Ovarian hormones can modulate the process of visceral pain perception and the susceptibility to stress.[87] Although younger children have not reached sexual maturity, this can apply to adolescents as well. However, when analyzing gender distribution at pre-pubertal age (≤10 years), there still was a persisting difference (boys 7.7% vs. girls 9.9%, OR 1.4, 59% CI 1.16, 1.79, *p*<0.001). Another reason might be the fact that females have a greater willingness to report somatic experiences, such as pain.[88] High pain profiles, indicating higher levels of pain and lower ability to cope with pain, were more often reported among girls.[89] Predominance of girls has been also described in other functional complaints, like functional constipation[90] and headache.[91,92]

In this systematic review, no association was found between age and prevalence of pediatric AP-FGIDs. Chitkara suggested a bimodal peak, between 4 and 6 year and preadolescence, in which the symptoms of abdominal pain are more prevalent.[1] More recent studies, however, showed a peak prevalence at adolescence.[23,24,27] Unfortunately due to great diversity in selected age groups among studies, we were unable to perform meta-analyses on single or narrow age groups and therefore we could not confirm these previous findings.

Epidemiological studies included in this SR showed that children with functional abdominal pain were significantly more often diagnosed with anxiety or depressive disorders compared to healthy children. Mechanisms and routes by which psychological factors affect functional abdominal pain are not fully known. Abdominal pain can cause psychological problems and conversely,[37,54] once developed abdominal pain and depression/anxiety may worsen each other. Moreover, both pain and symptoms of depression and anxiety can be the result of ineffective mechanisms of coping with stress, since low coping strategies are demonstrated in children with chronic abdominal pain.[93] Association of functional abdominal pain with stress and traumatic life events can be explained by unsuccessful coping styles as well. In addition, stressors have shown to be associated with enhanced visceral perception,[94] which is also described in pediatric IBS and RAP.[95,96] Increased responsiveness of central stress and arousal circuits and subsequently increase activity of the sympathetic nervous system can cause visceral hypersensitivity.[97]

Socioeconomic environment of the child has been reported to be a potential contributory factor to RAP.[47,75] Scandinavian studies have demonstrated that children living in low educated, low-income, worker families have higher levels of recurrent abdominal pain.[47,75] Our SR, however, reported that most studies conducted in Europe, Asia and US did not show any significant effect concerning the association between socioeconomic environment and functional abdominal pain. A recent well-conducted SR among adults, covering worldwide data, supports this latter finding.[98]

Strengths of the current study include a comprehensive and contemporaneous literature search that identified sufficient studies to allow pooling of data from almost 200,000 subjects. Because no language restrictions were applied, this is the first study which accomplishes all worldwide publications about the prevalence of pediatric functional abdominal pain. To date, a validated tool to assess the quality of epidemiological studies is lacking. Therefore a possible limitation of our study is the use of a self-made, not validated tool to assess the quality of the different epidemiological studies. A second limitation comes from the inclusion of studies using self-reported criteria for recurrent abdominal pain, since these criteria were not validated and less strict compared to the Apley and Rome criteria this can have distort the prevalence. However, our analyses showed the same prevalence rate in this case compared to the Apley and Rome II criteria. Interpretation of results was hampered by significant heterogeneity of included studies, due to methodological differences. To reduce this effect random effect models were used for meta-analyses. Publication bias was shown in some analyses, which can be eliminated by *trim and fill* method. However, because this method is known to perform poorly in the presence of substantial between-study heterogeneity, we decided not to correct our data by this method. Another limitation arises from the available studies and the reporting data within them. When calculating a pooled prevalence, there was a notable absence or ‘overrepresentation’ of studies conducted in certain geographical regions making it difficult to accurately estimate true global prevalence. For example, prevalence numbers from Turkey were only reflected by one small sample study. Lastly, information regarding associated factors was limited. Important data from studies comparing associated factors, such as psychosocial and socioeconomic factors, between AP-FGID-patients and controls were missing, because only prevalence studies were included.

In summary, functional abdominal pain occurs commonly worldwide. Female gender, psychological disorders, stress and traumatic life events increase the prevalence, while age and socioeconomic state are not associated. This high prevalence worldwide and its substantial impact on patients’ well-being justifies investment of resources and educational campaigns directed to prevention and optimal treatment, with special attention to psychological disorders and stress reduction.

## Supporting Information

S1 Appendix Quality assessment: criteria and outcome. (DOC)

S2 Appendix Forest plot overall prevalence. (DOC)

S1 PRISMA Checklist (DOC)

S1 Protocol (DOC)
