# Supplementary material for: Epidemiology of Pediatric Functional Abdominal Pain Disorders: A Meta-Analysis
Source: PLoS One. 2015 May 20;10(5):e0126982. doi: 10.1371/journal.pone.0126982 (PMC4439136; doi:10.1371/journal.pone.0126982)
Supplement: S1 Appendix — (DOC) [file pone.0126982.s001.doc]

***S1 Appendix 1. Quality assessment: criteria and outcome***

| **Quality assessment criteria** | | | | | | |
| --- | --- | --- | --- | --- | --- | --- |
| 1. Is method of subject selection described and appropriate? | | | | | | |
| 1. Are subject characteristics sufficiently described, i.e. do they match the target population regarding to gender and age? | | | | | | |
| 1. Is functional abdominal pain diagnosed appropriately? | | | | | | |
| 1. Are the survey instruments reliable and valid? | | | | | | |
| 1. Are the analytic methods described/justified and appropriate? | | | | | | |
| 1. Were the results reported in sufficient detail? | | | | | | |
| **Quality assessment outcome** | | | | | | |
| ***Study, year*** | ***1.*** | ***2.*** | ***3.*** | ***4.*** | ***5.*** | ***6.*** |
| Abu-Arafeh,(62) 1995 | 2 | 2 | 2 | 1 | 2 | 1 |
| Alfven,(42) 2008 | 1 | 1 | 2 | 1 | 2 | 1 |
| Apley,(3) 1958 | 2 | 2 | 2 | 1 | 2 | 2 |
| Bakoula,(74) 2006 | 2 | 2 | 2 | 2 | 2 | 1 |
| Bode,(51) 2003 | 2 | 2 | 2 | 1 | 2 | 1 |
| Boey,(52) 2003 | 2 | 2 | 2 | 1 | 2 | 1 |
| Boey,(56) 2001 | 2 | 2 | 2 | 1 | 2 | 1 |
| Boey,(57) 2001 | 1 | 2 | 2 | 1 | 2 | 2 |
| Boey,(60) 1999 | 2 | 1 | 2 | 1 | 2 | 1 |
| Brun,(46) 2007 | 2 | 2 | 2 | 2 | 2 | 1 |
| Christensen,(63) 1984 | 1 | 2 | 2 | 1 | 2 | 2 |
| Dalh,(73) 2005 | 1 | 2 | 2 | 1 | 2 | 2 |
| De Giacomo,(55) 2002 | 2 | 2 | 2 | 1 | 2 | 1 |
| Demirceken,(39) 2010 | 1 | 2 | 2 | 2 | 2 | 1 |
| Devanarayana,(30) 2012 | 2 | 2 | 2 | 2 | 2 | 2 |
| Devanarayana,(31) 2011 | 2 | 2 | 2 | 2 | 2 | 2 |
| Devanarayana,(32) 2011 | 2 | 2 | 2 | 1 | 2 | 2 |
| Devanarayana,(43) 2008 | 2 | 2 | 2 | 2 | 2 | 2 |
| Dong,(48) 2005 | 2 | 2 | 2 | 1 | 2 | 1 |
| Endo,(33) 2011 | 2 | 1 | 2 | 2 | 2 | 2 |
| Faull,(71) 1986 | 2 | 1 | 2 | 1 | 1 | 1 |
| Groholt,(75) 2003 | 2 | 2 | 2 | 1 | 2 | 2 |
| Gulewitsch,(22) 2013 | 1 | 1 | 2 | 2 | 2 | 2 |
| Harma,(54) 2002 | 2 | 1 | 2 | 1 | 2 | 1 |
| Helgeland, (37) 2010 | 2 | 1 | 2 | 1 | 2 | 1 |
| Hyams,(14) 1996 | 1 | 1 | 2 | 2 | 2 | 1 |
| Kokkonen,(69) 2004 | 2 | 1 | 2 | 1 | 2 | 1 |
| Liu,(34) 2011 | 1 | 2 | 2 | 1 | 2 | 2 |
| Lundby,(72) 1990 | 1 | 2 | 2 | 1 | 2 | 2 |
| Luntamo,(25) 2012 | 2 | 1 | 2 | 1 | 2 | 1 |
| Malaty,(67) 2007 | 1 | 2 | 2 | 1 | 2 | 2 |
| Mortimer,(70) 1993 | 2 | 2 | 2 | 1 | 2 | 2 |
| O'Donohoe,(61) 1996 | 1 | 2 | 2 | 1 | 2 | 2 |
| Oh,(50) 2004 | 2 | 2 | 2 | 1 | 2 | 1 |
| Ostberg,(47) 2006 | 2 | 2 | 2 | 1 | 2 | 1 |
| Park,(96) 2011 | 1 | 2 | 2 | 2 | 2 | 2 |
| Perquin,(59) 2000 | 2 | 2 | 2 | 1 | 2 | 1 |
| Petersen,(53) 2003 | 2 | 2 | 2 | 1 | 2 | 2 |
| Phavichitr,(26) 2012 | 1 | 2 | 2 | 2 | 2 | 1 |
| Rask,(40) 2009 | 2 | 1 | 2 | 1 | 2 | 1 |
| Reshetnikov,(58) 2001 | 2 | 2 | 2 | 2 | 2 | 1 |
| Romero,(23) 2013 | 2 | 2 | 2 | 1 | 2 | 1 |
| Sagawa,(24) 2013 | 2 | 1 | 2 | 2 | 2 | 2 |
| Saps,(21) 2014 | 1 | 2 | 2 | 2 | 2 | 1 |
| Sharrer,(64) 1991 | 2 | 2 | 2 | 1 | 2 | 2 |
| Silva,(35) 2011 | 2 | 2 | 2 | 1 | 2 | 1 |
| Sohrabi,(66) 2010 | 2 | 2 | 2 | 2 | 2 | 2 |
| Son,(44) 2008 | 1 | 1 | 2 | 1 | 2 | 2 |
| Song,(27) 2012 | 1 | 1 | 2 | 1 | 2 | 2 |
| Stanford,(45) 2008 | 2 | 1 | 2 | 1 | 2 | 1 |
| Telmesani,(41) 2009 | 1 | 1 | 2 | 1 | 2 | 1 |
| Tindberg,(49) 2005 | 1 | 2 | 2 | 1 | 2 | 1 |
| Uc,(68) 2006 | 1 | 2 | 2 | 2 | 2 | 1 |
| Youssef,(6) 2008 | 2 | 2 | 2 | 1 | 1 | 1 |
| Zheng,(28) 2012 | 1 | 2 | 2 | 2 | 2 | 1 |
| Zhou,(29) 2012 | 2 | 2 | 2 | 1 | 2 | 1 |
| Zhou,(36) 2011 | 2 | 2 | 2 | 1 | 2 | 2 |
| Zhou,(38) 2010 | 2 | 2 | 2 | 1 | 2 | 2 |
| *No=0 points; partial=1 point; yes=2 points* | | | | | | |
