# Supplementary material for: Epidemiology of Pediatric Functional Abdominal Pain Disorders: A Meta-Analysis
Source: PLoS One. 2015 May 20;10(5):e0126982. doi: 10.1371/journal.pone.0126982 (PMC4439136; doi:10.1371/journal.pone.0126982)
Supplement: S2 Appendix — (DOC) [file pone.0126982.s002.doc]

S3 Appendix 2. Forest plot overall prevalence
