# Supplementary material for: Epidemiology of Pediatric Functional Abdominal Pain Disorders: A Meta-Analysis
Source: PLoS One. 2015 May 20;10(5):e0126982. doi: 10.1371/journal.pone.0126982 (PMC4439136; doi:10.1371/journal.pone.0126982)
Supplement: S1 Protocol — (DOC) [file pone.0126982.s004.doc]

**Epidemiology of Paediatric Functional Abdominal Pain Worldwide; a Meta-analysis**

**Protocol**

**Questions:**

- What is the worldwide prevalence of functional abdominal pain?
  - Distribution to age, gender and geographic?
  - Wat is de verdeling naar subtypes van AP-FGID (IBS, FAP(S), FD, AM)?
- Which factors are associated with functional abdominal pain?
  - Family history
  - Psychological factor
  - Socio-economic factors
  - Quality of life
  - life-events; sexual abuse

**Inclusion criteria:**

1. children aged 4-18 yrs
2. functional abdominal pain according to the ROME I, II, III criteria, Apley and Naish criteria or defined by the presence of nonorganic abdominal pain in children qualified by at least three episodes of abdominal pain, weekly episodes of abdominal pain, and/or a symptom duration of at least 3 months
3. epidemiology studies of birth cohort, school based and general population samples
4. results reported on the epidemiology, prevalence or incidence in children with functional abdominal pain

**Exclusion criteria:**

1. case reports
2. studies that had entry criteria that limited the generalizability of the findings, such as evaluation of only patients with specific co-morbidities or reports of individuals who had sought health care for their symptoms.

**Databases:** Medline, Embase, Cinahl and PsychInfo

**Search**

PUBMED

fgid[tiab] OR functional gastrointestinal disorder*[tiab] OR irritable bowel syndrome[mh] OR irritable colon[tiab] OR ibs[tiab] OR irritable bowel[tiab] OR ((functional[tiab] OR recurr*[tiab] OR chronic[tiab]) AND abdominal pain[tiab]) OR functional dyspepsia[tiab] OR abdominal migraine[tiab] OR abdominal epilepsy[tiab] OR stomach ache[tiab]) AND (child OR childr* OR childh* OR preschool OR schoolchild* OR schoolage* OR adolescent OR adoles* OR teen* OR youth* OR minor* OR underage* OR juvenil* OR boy OR boys OR girl* OR puber* OR pediatric* OR pediatric OR paediatric* OR primary school* OR elementary school* OR high school* OR kindergart*) AND (epidemiology OR incidence OR prevalence)

EMBASE

1. (figd or (functional adj3 gastrointestinal disorder*) or ibs or irritable bowel or ((functional or recurr* or chronic*) adj5 abdominal pain) or (functional adj3 dyspepsia) or stomach ache or abdominal epilepsy or (abdominal adj3 migraine)).tw.

2. exp Irritable colon/

3. 1 or 2

4. (child* or boy or boys or girl* or prepube* or pubert* or pubesc* or adolescen* or juvenil* or minors or youth* or teen* or p?ediatric*).tw.

5. adolescent/ or juvenile/ or boy/ or girl/ or puberty/ or prepuberty/ or pediatrics/ or primary school/ or high school/ or nursery school/ or school/

6. 4 or 5

7. epidemiology/ or morbidity/ or mortality/

8. (incidence or prevalence).tw.

9. ep.fs.

10. 7 or 8 or 9

11. 3 and 6 and 10

PsycInfo

1. (figd or (functional adj3 gastrointestinal disorder*) or ibs or irritable bowel or ((functional or recurr* or chronic*) adj5 abdominal pain) or (functional adj3 dyspepsia) or stomach ache or abdominal epilepsy or (abdominal adj3 migraine)).tw.

2. exp Irritable Bowel Syndrome/

3. 1 or 2

4. ("160" or "180" or "200").ag.

5. (child* or boy or boys or girl* or prepube* or pubert* or pubesc* or adolescen* or juvenil* or minors or youth* or teen* or p?ediatric*).tw.

6. 4 or 5

7. 3 and 6

8. epidemiology/ or morbidity/ or mortality/

9. (incidence or prevalence).tw.

10. 8 or 9

11. 7 and 10

CINAHL

| **#** | **Query** |
| --- | --- |
| S17 | S12 AND S16 |
| S16 | S13 OR S14 OR S15 |
| S15 | AB epidemiology or incidence or prevalence or morbidity or mortality |
| S14 | TI epidemiology or incidence or prevalence or morbidity or mortality |
| S13 | (MH "Epidemiology+") |
| S12 | S7 OR S11 |
| S11 | S6 AND S10 |
| S10 | S8 OR S9 |
| S9 | AB child OR childr* OR childh* OR preschool OR schoolchild* OR schoolage* OR adolescent OR adoles* OR teen* OR youth* OR minor* OR underage* OR juvenil* OR boy OR boys OR girl* OR puber* OR pediatric* OR pediatric OR paediatric* OR primary school* OR elementary school* OR high school* OR kindergart |
| S8 | TI child OR childr* OR childh* OR preschool OR schoolchild* OR schoolage* OR adolescent OR adoles* OR teen* OR youth* OR minor* OR underage* OR juvenil* OR boy OR boys OR girl* OR puber* OR pediatric* OR pediatric OR paediatric* OR primary school* OR elementary school* OR high school* OR kindergart |
| S7 | S1 OR S2 OR S3 OR S4 OR S5  Beperkingen - Leeftijdgroepen: Child, Preschool: 2-5 years, Child: 6-12 years, Adolescent: 13-18 years |
| S6 | S1 OR S2 OR S3 OR S4 OR S5 |
| S5 | AB (functional or recurr* or chronic*) N5 abdominal pain |
| S4 | TI (functional or recurr* or chronic*) N5 abdominal pain |
| S3 | AB figd or (functional N3 gastrointestinal disorder*) or irritable colon or abdominal migraine or stomach ache or abdominal epileps* |
| S2 | TI figd or (functional N3 gastrointestinal disorder*) or irritable colon or abdominal migraine or stomach ache or abdominal epileps* |
| S1 | (MH "Colonic Diseases, Functional+") |

**Data collectie**

- 1. author
  2. year
  3. location
  4. age cohort
  5. population
  6. sample size
  7. method of data-collection (questionnaires)
  8. criteria for functional abdominal pain
  9. prevalence functional abdominal pain
  10. prevalence subtypes (FD, IBS, FAP, FAPS, AM)
  11. prevalence sexes (F, M)
  12. prevalence age groups
  13. prevalence geographic area
  14. prevalence ethnic groups
  15. prevalence rural/urban
  16. Family history FAP/IBS
  17. Psychosocial aspects (depressive symptoms, anxiety, behavioural problems)
  18. Socio-economic state (highest education parents, family income)
  19. Quality of life
  20. school absence
  21. life-events

**Quality assessment**

YES(2) PARTIAL(1) NO(0)

1. Is method of subject selection described and appropriate?

2. Are subject characteristics sufficiently described? And do the match the target population?

3. Was functional abdominal pain diagnosed appropriately?

4. Are the survey instruments reliable and valid?

5. Was the analytic methods described/justified and appropriate?

6. Results reported in sufficient detail?

***Referenties Quality assessment:***

1. Boyle MH. EBMH NOTEBOOK Guidelines for evaluating prevalence studies. Evid Based Mental Health *1998;1:37-39*

# 2: http://pediatrics.aappublications.org/content/125/4/768.full.pdf+html?sid=5ddefb0a-9d7b-4003-8630-28fe6b0f99f3

*3. Kmet LM, Lee RC, Cook LS. Standard quality assessment criteria for evaluating primary research paper from a variety of fields.AHFMR, HTA Initiative 2004;13:1–22.*
